# Spin-informed universal graph neural networks for simulating magnetic ordering

**DOI:** 10.1073/pnas.2422973122

**Published:** 2025-07-01

**Authors:** Wenbin Xu, Rohan Yuri Sanspeur, Adeesh Kolluru, Bowen Deng, Peter Harrington, Steven Farrell, Karsten Reuter, John R. Kitchin

**Affiliations:** ^a^National Energy Research Scientific Computing Center, Berkeley, CA 94720; ^b^Lawrence Berkeley National Laboratory, Berkeley, CA 94720; ^c^Department of Chemical Engineering, Carnegie Mellon University, Pittsburgh, PA 15213; ^d^Fritz-Haber-Institut der Max-Planck-Gesellschaft, Berlin D-14195, Germany

**Keywords:** uMLIP, magnetic materials, magnetic ordering, data-centric AI

## Abstract

The screening and discovery of magnetic materials are hindered by the computational cost of first-principles density-functional theory (DFT) calculations required to find the ground state magnetic ordering. Although universal machine-learning interatomic potentials (uMLIPs), also known as atomistic foundation models, offer high-fidelity models of many atomistic systems with significant speedup, they currently lack the inputs required for predicting magnetic ordering. In this work, we present a data-efficient, spin-informed graph neural network framework that incorporates spin degrees of freedom as inputs and preserves physical symmetries, extending the functionality of uMLIPs to simulate magnetic orderings. This framework speeds up DFT calculations through better initial guesses for magnetic moments, determines the ground-state ordering of bulk materials and even generalizes to magnetic ordering in surfaces. Furthermore, we implement a closed-loop anomaly detection approach that effectively addresses the classic “chicken-and-egg” problem of creating a high-quality dataset while developing a uMLIP, unearthing anomalies in large benchmark datasets and boosting model accuracy.

## Significance

The development of universal machine-learning interatomic potentials capable of simulating magnetic ordering is vital for the in silico discovery of indispensable magnetic materials across vast chemical spaces. To date, progress has been hindered by challenges in model design and the availability of high-quality datasets. Here, we introduce a general spin-informed graph neural network framework, coupled with an anomaly detection approach, that achieves state-of-the-art performance in simulating magnetic ordering and enhances the quality of large benchmark datasets. These developments broaden the capabilities of atomistic foundation models and advance the evolution of data-centric AI.

The urgent need to tackle climate change has induced a massive shift in the economy toward electrification, with the automobile industry being among the early adopters (1). Beyond electric vehicles, wind turbine generators (2), hard disk drives, MRI machines, consumer electronics (3) and robotics systems for automation (4) all hinge on magnetic materials. The discovery and development of corresponding magnetic materials require reliable high-throughput methods to efficiently explore the vast design space of the periodic table. The surge in electrification is projected to put severe strain on the supply of raw materials that eventually make their way into permanent magnets.

Chemical intuition-based methods, such as the Goodenough-Kanamori-Anderson rules (5) and bond valence analyzer (6), often fail to identify important magnetic materials, like inverse spinels (7), due to their limited accuracy. In contrast, traditional density-functional theory (DFT) methods, while accurate, are hampered by high computational costs. Machine-learning interatomic potentials (MLIPS), and in particular their universal MLIPS (uMLIPS), which exhibit transferability across the periodic system of elements, have emerged as powerful tools for discovering nonmagnetic materials, offering a favorable balance between accuracy and computational efficiency. Extending these methods to magnetic materials would be highly desirable (8).

However, there is a classic “chicken-and-egg” problem of creating a high-quality dataset while developing a uMLIP. On one hand, the quality of the data on which AI models are trained determines how effective those models can be (9). Existing large benchmark databases are predominantly composed of consistent samples that have taken years to populate. Despite considerable efforts, inconsistent labels still persist due to a lack of convergence. The challenging task then becomes to identify those minority samples that show anomalous feature–label relationships: unearthing the needles in the haystack. On the other hand, AI models can introduce a valuable inductive bias, ensuring that feature representations are correlated with the labels (10). This makes it possible to single out the minority anomalies in the dataset and to refine it by recomputing those samples under more scrutinous settings. Alternatively, and as we show in this work, we can also exploit the embeddings generated by our trained uMLIP within a clustering algorithm to automate anomaly detection and dataset refinement. Implementing this as a dataset refinement loop, in turn, results in more accurate models.

The development of MLIPs has made substantial progress in addressing specific classes of magnetic materials, employing both descriptor-based (11–13) and neural network-based approaches (14–19). For modeling collinear magnetic systems, Yu et al. (16) introduced Heisenberg edge graph neural networks (GNNs) and spin-distance edge GNNs (SEGNNs), where the former leverages the dot product of spin vectors in the Heisenberg model to learn two-body magnetic interactions, while the latter uses it as edge features in GNNs to capture higher-order magnetic interactions. However, the expressivity of these models is limited by their reliance on the spin vector in the form of a dot product. Hu et al. (20) developed a collinear magnetic potential based on DeepSPIN (19), explicitly introducing “pseudo-atoms" to describe spin–lattice interactions. This approach significantly increases the number of nodes and edges in the graph, leading to computational overhead that can be prohibitive in the context of expensive graph convolution operations. Behler et al. (14) presented spin-dependent atom-centered symmetry functions as input for high-dimensional neural network potentials (HDNNPs), but this method cannot handle complex spin configurations such as ferrimagnetic (FiM) ordering.

In an attempt to develop a charge-informed uMLIP, CHGNet (18) incorporated magnetic moments as the proxy for atomic valence states. However, the model predicts an averaged potential energy surface over different magnetic configurations, limiting its usefulness in addressing magnetic ordering. Given the inextricable correlation between the atomic spin degree of freedom and the electronic energy (21, 22), it is likely that a model that solely incorporates the magnitude of magnetic moments as a regularizer in the loss function fails to describe the electronic energy that is sensitive to spin configurations. In the same vein, Sanspeur et al. (7) highlight this as a deficiency of their magnetic moment predictions, which can only handle the magnitude and not the ordering.

In this work, we introduce a spin-informed general GNN framework that not only achieves state-of-the-art prediction accuracy but also incorporates an anomaly detection approach to flag outliers in large benchmark datasets, effectively addressing the classic “chicken-and-egg” problem. By leveraging the unique capability of our proposed GNN framework to identify outliers, one can recompute these outliers to enhance the quality of the dataset, then retrain the model to flag even more outliers as part of an active learning cycle. To demonstrate the state-of-the-art capabilities in simulating magnetic ordering, and consistent with prior research (7), we confirm that our model can be useful at providing optimal initial guesses for the magnitude of the magnetic moments to electronic structure codes. We show that the model can effectively rank the energies of collinear magnetic orderings at the level of the bulk. More interestingly, we show that the model also generalizes to surfaces, where the combinatorics become more acute. We demonstrate how the model can be used to optimize the spin-ordering degrees of freedom in a slab and, given the cascading effects of a poor initial guess for the magnetic configuration, how that can have significant effects on standard catalytic descriptors like adsorption energies, consistent with recent work (23), as well as reveal adsorbate-induced nonlocal spin effects.

## Results

### Spin-Informed GNN Framework.

In the realm of MLIPs, a “forward pass” is analogous to a DFT single-point calculation and allows us to bypass Self-Consistent-Field (SCF) calculations, which predict the properties for given atomic positions and element identities. For spin-polarized DFT calculations, an initial guess for the atomic magnetic moments is required. Due to the deterministic nature of DFT, under appropriate computational settings, magnetic moments converge from initial guesses through a many-to-one mapping during the SCF cycle. Inspired by this, we introduce a general spin-informed GNN framework that incorporates an initial guess (scalar) and an atomic spin coordinate (unit vector). We demonstrate the use of this framework in connection with two baseline GNN models from the Open Catalyst Project (24, 25): SchNet (26) and GemNet-OC (27). Note that Behler et al. (14) applied a similar atomic spin coordinate into HDNNPs. However, relying solely on a unit spin vector is insufficient to distinguish between AFM and FiM orderings for the same atomic structure without the aid of an initial guess (scalar).

**Fig. 1.**
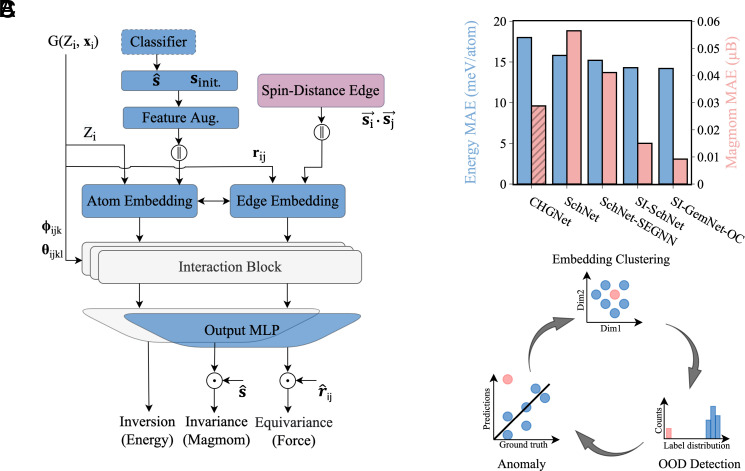
(*A*) Main components of the Spin-informed GNN architecture using GemNet-OC as the backbone. Modifications in SI-GemNet-OC relative to the original GemNet-OC are highlighted in blue. Spin-distance edge methods, highlighted in purple, are adapted from ref. 16 and used exclusively in SEGNNs for comparison. Inversion refers to the spin-inversion symmetry in energy prediction related to spin configurations. Invariance pertains to magnetic moment (magmom) predictions, while equivariance is concerned with the direct force prediction with respect to atomic positions. The symbol ‖ denotes concatenation. (*B*) Performance evaluation of Spin-informed GNNs compared to state-of-the-art uMLIPs. The energy and magmom MAE are shown in blue and purple. The stripe pattern on CHGNet indicates that it predicts only the magnitude of magmoms, while the other models consider both the sign and magnitude. (*C*) Schematic of the anomaly detection workflow.

Fig. 1*A* presents the Spin-Informed GemNet-OC (SI-GemNet-OC) architecture. GemNet-OC (27), an enhanced variant of GemNet (28), is a GNN that integrates geometric information into its message-passing process. This geometric information includes distances between atoms ($r_{\mathrm{ij}}$), angles between neighboring edges ($\phi_{\mathrm{ijk}}$), and dihedral angles formed by triplets of edges ($\theta_{\mathrm{ijkl}}$). The message-passing process relies on two key components: atom embeddings and edge embeddings. These embeddings are updated based on the geometric information, and property predictions are subsequently derived from these updated embeddings. We also tested the recently developed general spin-distance edge GNN framework (16), which uses the spin dot product as an input and is implemented based on edge embeddings highlighted in purple in Fig. 1*A*. Our primary design focus is the incorporation of the spin degrees of freedom and the preservation of physical symmetry within the architecture. Initial guesses and atomic spin coordinates are introduced into the atom embeddings, while physical symmetry is maintained at the prediction level. Additional details on these design focuses and SI-GemNet-OC are provided in *Methods*.

It is crucial to emphasize the distinct physical symmetries associated with spin degrees of freedom in noncollinear vs. collinear magnetic systems. In a typical DFT setup, noncollinear systems assign on-site magnetic moments specified by each atom’s position, resulting in a subtle interplay between SO(3) symmetry transformations for geometric tensors and SU(2) transformations for spinors. In contrast, collinear systems define a global spin axis that is independent of atomic positions, allowing the symmetry transformations of atomic positions and spins to be treated separately. Failing to account for this distinction can significantly increase model complexity and necessitate a training dataset that is orders of magnitude larger. Further details on the incorporation of symmetries are provided in *Methods*.

### Performance Evaluation on Materials Project Database.

**Fig. 2.**
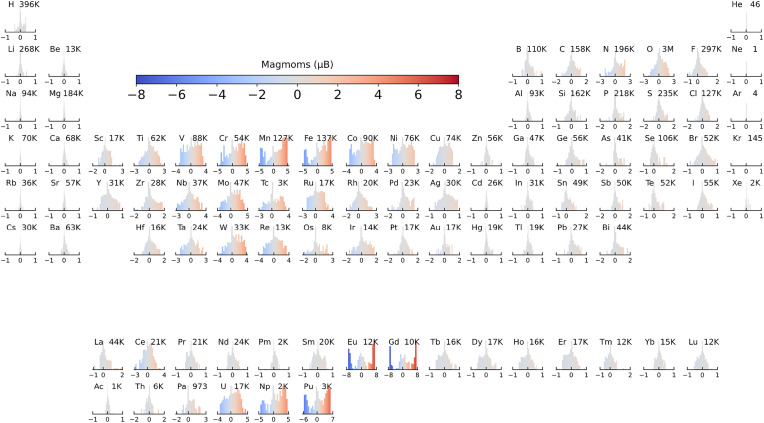
Distribution of atomic magnetic moment (magmom) labels in the MP-MM dataset, projected onto each element across the periodic table. The color bar indicates the range of atomic magnetic moments in *μ*_B_. The chemical symbol of each element is shown in the *Upper Left* corner of each subplot, while the number of data points (counts) is displayed in the *Upper Right* corner. The distribution of magmom labels across different elements is highly imbalanced, where 3d, 4d, and 5d transition metals, along with a few rare-earth elements such as Eu, Gd, Np, and Pu, exhibit relatively broader distributions. In contrast, many other elements span a much narrower range.

The Materials Project Trajectory (MPtrj) dataset (18), one of the largest inorganic materials datasets, was used in this study with a view to capturing the relationship between magnetic configurations and energetic stability. Only those structures with magnetic moment (magmom) labels were extracted. This subset is termed the MP-Magnetic Materials (MP-MM) dataset (see *Methods* for dataset description and preprocessing). Overall, the MP-MM dataset contains 284,110 atom configurations with a total of 7,941,047 magmom labels. The distribution of magmom labels across different elements is presented in Fig. 2. It can be seen that the magmom labels are highly imbalanced compared to energy labels (*SI Appendix*, Fig. S1). The majority of magmom labels collapse to a local magnetization of approximately 0 *μ*_B_, with magmoms in the range of −0.1 to 0.1 *μ*_B_ accounting for 87.2% of the total dataset.

We trained two SI-GNN models, SI-SchNet and SI-GemNet-OC, on the MP-MM dataset using a random 8:1:1 split for the training, validation, and test sets where only magmom and energy labels were included. Fig. 1*B* presents the results on test set in comparison to a state-of-the-art uMLIP, CHGNet, which is also capable of predicting the magmom label. Between SchNet and its variants, SchNet-SEGNN shows a considerable improvement of approximately 27% in magmom predictions compared to the standard SchNet model, due to the inclusion of magmom information in the spin-edge distance. Meanwhile, SI-SchNet demonstrates a significant improvement of about 73%, achieving a Mean Absolute Error (MAE) of 0.015 *μ*_B_. The inferior performance of SchNet-SEGNN relative to SI-SchNet can be attributed to its lower expressivity when leveraging the spin vector in the form of a dot product. Ultimately, with the introduction of geometric message passing and quadratic iteration, SI-GemNet-OC further reduces the MAE to 0.009 *μ*_B_.

Relative to CHGNet (18), which achieved a MAE of 0.029 *μ*_B_ for magnetic moment predictions, SI-GNN exhibits significantly lower error. This improvement is due to the explicit incorporation of spin information, which is crucial for breaking the symmetry of embeddings necessary to capture spin effects. It is important to note that our approach aims to predict both the sign and magnitude of magmoms, whereas CHGNet only predicts the magnitude, making it inherently unable to distinguish between different magnetic orderings. For energy prediction, all models demonstrate reasonably low energy MAEs below 20.0 meV/atom, indicating substantial potential to study the energetics of magnetic materials. Furthermore, the inclusion of spin information allows SchNet-SEGNN and SI-SchNet to reduce the energy MAE to 15.2 meV/atom and 14.3 meV/atom, respectively, compared to the standard SchNet (15.8 meV/atom). SI-GemNet-OC achieves a slightly lower-energy MAE of 14.2 meV. The parity hexbin plot of DFT-calculated vs. ML-predicted energies is presented in *SI Appendix*, Fig. S4.

The initial guesses in the MP-MM dataset include both MP and non-MP initializations, with the latter taken from the last frame of the first relaxation trajectory in a double-relaxation scheme (*Methods*). Our tests show that non-MP initializations lead to improved predictive performance, highlighting the benefits of better initial guesses for the SI-GNN model (*SI Appendix*, Fig. S7). To train a MLIP that reliably captures the many-to-one mapping in collinear magnetic systems, initial guesses should be drawn from a more diverse set rather than relying solely on MP initialization. This is analogous to the fact that a robust classifier requires diverse input to ensure generalizability. In the MP dataset, MP initialization is highly sparse, containing only a single or a few element–value pairs, where the latter refers to elements in different oxidation/ionic states, and thus provides insufficient coverage. The inclusion of non-MP initializations in the current MP-MM dataset helps alleviate this problem. Furthermore, we systematically explore the effect of different types of initial guesses as inputs to the SI-GNN models, as presented in *SI Appendix*, Table S1, and provide practical guidance in *SI Appendix*, section S2.3.

**Fig. 3.**
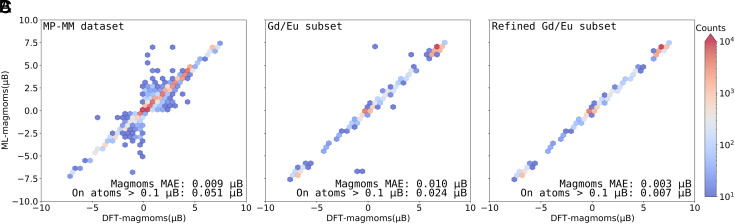
Parity hexbin plots of DFT-calculated vs. ML-predicted magmoms using SI-GemNet-OC for: (*A*) test set of the MP-MM dataset: The fact that the majority of the data points for the test set lie on the parity line suggests that there are sufficient high-quality training data for the model to learn the inherent patterns that map the magnetic ordering to the electronic energy. Large deviations from the parity line would most likely stem from an inconsistency between the label and the features, which we can corroborate with the anomaly detection algorithm, (*B*) training set of the Gd/Eu subset, (*C*) training set of the refined Gd/Eu subset via anomaly detection. The color bar denotes the number of points in each hexbin, and only hexbins with more than 5 points are displayed.

Given that the majority of magmom labels collapse to a local magnetization of ∼0 *μ*_B_, an overall MAE of 0.009 *μ*_B_ may not accurately reflect the predictive performance for magnetic atoms, as errors could be masked by averaging across a substantial number of nonmagnetic atoms. Fig. 3 presents a hexbin plot of ML-predicted vs. DFT-calculated magmoms where only atoms with magmoms greater than 0.1 *μ*_B_ are shown. It is not surprising that the MAE goes from 0.009 *μ*_B_ to 0.051 *μ*_B_, with the less biased metric nevertheless still reflecting an accurate model. More intriguingly, while most predictions align along the diagonal, indicating high accuracy, a considerable number of outliers (859 data points with error greater than 1 *μ*_B_) are scattered within the range of −2.5 to 2.5 *μ*_B_, and a similar pattern is also observed in the training hexbin plot (*SI Appendix*, Fig. S5). Notably, some groups exhibit an MAE as high as 6.5 *μ*_B_. Closer inspection reveals that these groups are Gd/Eu-based compounds. It is important to highlight that Gd-based magnetic materials are a highly valued class of materials known for their unique magnetic properties and their use in various advanced technological applications (29, 30). This motivates us to zero in on a Gd/Eu subset.

### Unveiling Data Quality Issues.

To investigate the presence of outliers and establish a robust ML model, the Gd/Eu subset was extracted from MP-MM. The subset includes 6,896 atom configurations with a total of 128,519 magmom labels, with additional details including the distribution of magmoms and energy provided in *Methods* and *SI Appendix*, Fig. S2. We then trained SI-GemNet-OC on this subset using a 90/10 random split for training and validation. Large outliers can be observed in both the training and validation parity plots (*SI Appendix*, Fig. S6). The training parity plot suggests that the ML model is either not expressive enough or that there are anomalous samples present, which the model cannot fit well. In fact, the high data imbalance within this subset, with only a minimal fraction of labels around −1 and 1 *μ*_B_, indicates the potential presence of mislabeled or anomalous samples.

Inspired by this, we then attempted to refine this subset by extracting those 234 samples having magmoms in the range of −2 to 2 *μ*_B_. We then performed DFT calculations with an initialization of 7 *μ*_B_ magmoms on Gd/Eu. As shown in *SI Appendix*, Fig. S3, a clear shift in the distribution can be seen from around −1 and 1 to −7 and 7 *μ*_B_, indicating that the previous subset suffered from data quality issues where the DFT calculations were not well converged (see *Applications* for further details). With this refined dataset, the resulting parity plot indeed showed significant improvement, as illustrated in Fig. 3*B*.

The remaining outliers highlight issues arising either from the inappropriate description with the Perdew–Burke–Ernzerhof (PBE) functional for this class of materials or from the suboptimal DFT settings themselves. This necessitates the use of an anomaly detection method to further refine the dataset, ultimately leading to a more robust ML model. We developed a method that integrates a clustering algorithm with out-of-distribution (OOD) detection (see Fig. 1*C* for a schematic illustration). In practice, we extracted atom embeddings from the pretrained model and applied a Gaussian mixture model (GMM) to cluster the samples based on the similarity of the learned embeddings. We then perform an OOD detection using a z-score method for each group (*Methods*). It is evident in *SI Appendix*, Fig. S9 that the anomalous samples stand out with large deviations from the domain samples in each group, thus effectively identifying them in the training dataset.

**Fig. 4.**
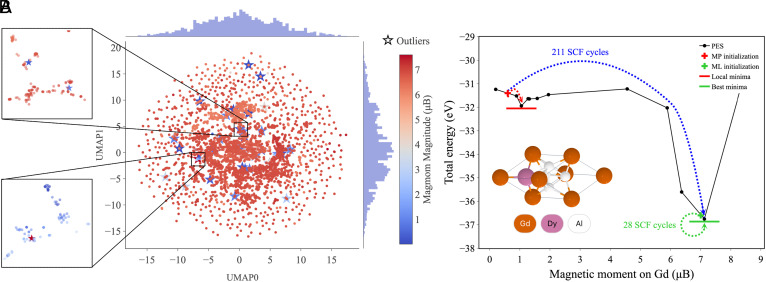
(*A*) Two-dimensional projection of the 256-dimensional atom embeddings extracted from the SI-GemNet-OC model on the training set of the Gd/Eu subset. The dimensional reduction is solely performed on Gd and Eu atom embeddings. The abundance of red points suggests that most magmom labels (magnitudes) are ∼7 *μ*_B_. Star markers highlight large outliers with errors greater than 3 *μ*_B_. In the topmost *Inset*, when we zoom into the 2-dimensional embedding space, we see that there are atoms whose magmom magnitudes are ∼1 *μ*_B_ but that their representation lies in a pool of nonoutlier atoms whose magnitudes are predominantly ∼7 *μ*_B_. Similarly, the *Bottom Inset* shows that when we zoom into the 2-dimensional embedding space, we see that there are atoms whose magmom magnitudes are ∼7 *μ*_B_ but that their representation lies in a pool of nonoutlier atoms whose magnitude are predominantly ∼1 *μ*_B_. These outlier labels are inconsistent. (*B*) Schematic of the PES that we probed for GdDyAl^4^ by enforcing specific spin configurations using the NUPDOWN parameter in VASP (32–34) and a converged single point. The corresponding task ID in Materials Project is mp−1785039. The MP initialization together with a tricky PES landscape leads to convergence to a suboptimal local minima around 1 *μ*_B_, which results in outliers in Fig. 4*A*.

To better illustrate this, the extracted atom embeddings were projected into 2D space using UMAP (31). As seen in Fig. 4*A*, samples with similar magmom labels generally tend to group together in the embedded latent space (35, 36). However, the large outliers (star markers) exhibit labels that are very distant within their group, indicating anomalous samples. This occurs for anomalies with both small and large magmom magnitudes (see the *Inset* figures in Fig. 4*A*). The hypothesis is that there are more good labels than bad ones; given the large parameter space, the model should be able to fit the data and produce embeddings that can inform the label. It will not be able to do so for samples that are embedded in a way inconsistent with the training label. Finally, by removing these anomalous samples identified through our anomaly detection method and retraining the SI-GemNet-OC model, we achieve a significantly better fit (Fig. 3*C*), implying a much more robust ML model.

In addition, we also tested the anomaly detection method to another Cr subset that does not contain f-electrons. The results are highly consistent with those from the Gd/Eu subset, showing similarly improved predictive accuracy and no significant outliers (see *SI Appendix*, Fig. S8 for the resulting parity plot). Intriguingly, the proportion of anomalies identified is 0.7% in the Gd/Eu subset and 0.8% in the Cr subset. This closely aligns with the proportion of large outliers (error > 1.5 *μ*_B_) in the test split of the full dataset, which is approximately 0.9%. These findings suggest that the scope of the anomaly issue in the MP dataset accounts for roughly 1% of the data.

### Applications.

In this section of the paper, we illustrate potential use cases for the spin-informed ML potential described above. We demonstrate and rationalize potential computational efficiency gains from good initial guesses for the magnitude of the magnetic moment in a rare-earth magnetic material (GdDyAl^4^), which exhibits two prominent local minima. Subsequently, we show that the incorporation of spin features in the models unlocks the ability to correctly rank the bulk energetic stability of magnetic orderings when applied to a dataset curated from previous work (37). This reveals that a large proportion of materials initialized in the ferromagnetic (FM) ordering converge to suboptimal local minima on the potential energy surface. Finally, we show that the spin-informed ML potential, which was solely trained on bulk data, is also able to extrapolate to surface structures and optimize their energy as a function of the spin configuration in such permutationally challenging structures.

While the utility of our spin-informed model in practical computational chemistry settings is self-evident, active learning dataset refinement strategies like those described in *Methods* are poised to improve its performance over time.

#### Suboptimal converged structures from poor initial guesses and a tricky PES.

Given the local nature of the electronic minimization routine with spin polarization in prevalent DFT packages like the VASP package employed herein (32–34), the initial guess for the magnetic moments plays an important role in the type of converged structure that one can access (7). In this section, we rationalize, using one of the outlier samples whose PES we probed in Fig. 4*B*, how one can end up with suboptimal converged structures from poor initial guesses.

If one is lucky, a poor guess can still escape a suboptimal minimum during the SCF cycle but it will take longer to converge to the optimal minimum. In the case of GdDyAl^4^, where the only strongly magnetic atom is Gd, we see from Fig. 4*B* that an initialization for the Gd atom around 7 *μ*_B_ should allow convergence to the optimal minimum in the least amount of SCF steps. We confirm that in VASP, initializing the Gd ion with 7 *μ*_B_ (as predicted by our SI-GNN models) converges to the superior minimum in 28 SCF cycles. In contrast, initializing the Gd ion with 0.6 *μ*_B_ (as was the case in the MP database) still ends up converging to 7 *μ*_B_, in the majority of cases, but costs 211 SCF cycles. On one hand, this underscores the usefulness of ML initialization in accelerating DFT calculations. On the other hand, this also illustrates why a challenging PES landscape can result in atomic embeddings and magnetic moments that are insidiously inconsistent (*Unveiling Data Quality Issues*): If one initializes the magnetic moment on the Gd ion at 0.6 *μ*_B_ and sets the maximum number of SCF steps to 100, then one ends up with a suboptimal magnetic state (around 1 *μ*_B_). This also underscores the importance of automated convergence checks in high-throughput DFT efforts (38). In addition, based on the PES of GdDyAl^4^, we demonstrate the many-to-one mapping between initial guesses and converged magnetic moments using SI-GemNet-OC. All 12 initial guesses yield predictions around 7.15 *μ*_B_, closely aligning with the DFT-calculated minimum of 7.12 *μ*_B_ (*SI Appendix*, Fig. S12).

#### Bulk ordering prediction.

**Fig. 5.**
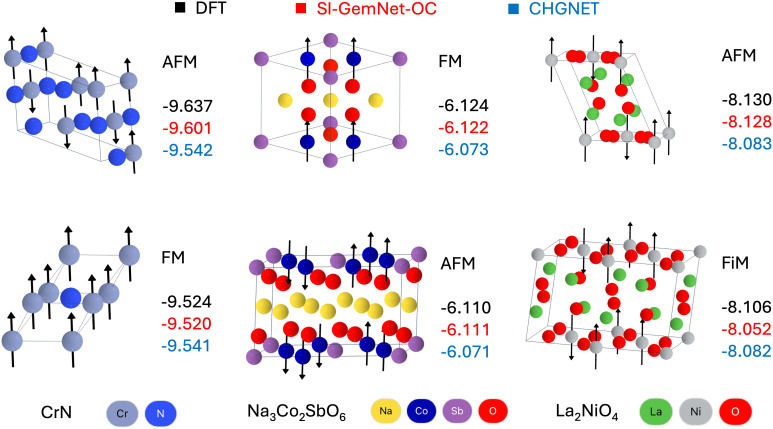
Validating the ability of the model to energetically rank the magnetic orderings of 3D materials. FM, AFM, and FiM are ferromagnetic, antiferromagnetic, and ferrimagnetic ordering, respectively. The magnetic moments are depicted with black arrows. Energy calculated by DFT is in black. Energy predicted by SI-GemNet-OC and CHGNet are in red and blue. The unit of energy is eV/atom. Their corresponding task IDs in Materials Project are mp−1232726, mp−1232909, mp−1786123, mp−1232537, mp−1232479, and mp−1232766, respectively. We would only trust the model to energetically rank magnetic orderings whose energy differences are greater than its energy MAE.

While the predicted magnitudes are thus valuable for the initialization of spin-polarized DFT calculations, the significant contribution of our model lies in its ability to also capture the direction of the magnetic moment and thereby any magnetic ordering and its relation to the energetic stability. In order to validate our model, we have established a holdout dataset originating from ref. 37, where the magnetic orderings have been enumerated and their associated energetics computed with DFT. There are only ∼486 structures in this dataset and a large proportion of them are very close in energy, which makes the case for running such magnetic ordering enumeration high-throughput DFT workflows at larger scales for the purpose of generating richer training data. Fig. 5 illustrates some examples where the model is able to successfully rank the magnetic ordering based on their energetic stability. In contrast, CHGNet predicts nearly identical energetics for different magnetic orderings, since only the averaged potential energy over different magnetic configurations is captured. The average Spearman’s correlation coefficient for ranking magnetic orderings is 0.896 across 64 groups comprising 486 materials, with a 10 meV/atom threshold applied, where energy differences within this range are assigned the same rank. The rest of the predictions are available at this Zenodo link.

#### Surface ordering prediction.

Finally, we test whether our model, which was only trained on bulk level data, can be useful at the surface level, where the combinatorics become even more daunting due to broken symmetry and the involvement of more atoms.

We cleave two bulk materials [Fe_2_O_3_ and CrN (39)] along the (110) and (100) planes, respectively. The assumption that we make in placing the initial magnetic moments on the slabs is that they inherit the same ordering as that in the bulk, which is a common assumption in catalysis. The model correctly ranks antiferromagnetic (AFM) ordering as the most stable configuration at the bulk level for both materials. The model also ranks the AFM initialized slabs as more stable than their FM counterparts, which we validated by explicit DFT calculations.

**Fig. 6.**
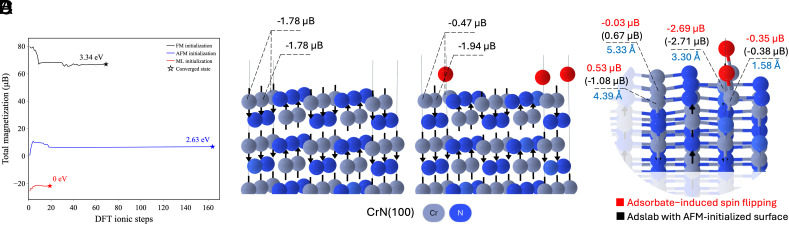
Spin-informed ML potential is used as a surrogate in Monte Carlo spin flipping for surface-related applications. (*A*) Plot of total magnetization in *μ*_B_ vs. ionic steps index for different magnetic ordering initializations of CrN slab. The ordering initialization from the ML simulation sees less fluctuations and converges in fewer ionic steps than the AFM or FM ordering initializations and most importantly converges to a significantly lower-energy minimum. (*B*) CrN was cut along the (100) plane. Cr is depicted in pale blue and N in dark blue. The most stable ordering found from the ML-potential-aided simulation is FiM, since the total magnetization of the unit cell is not zero. Most of the magnetism is at the level of the Cr ions. (*C*) Nitrogen adsorbate at Cr vacancy site for the most stable slab ordering identified by our spin-informed ML potential. The Nitrogen adsorbate is highlighted in red. Structure and magnetic ordering rendered with crystal toolkit (40), with slight modifications to internal settings for easier visualization. (*D*) Adsorbate-induced spin flipping (red) compared to the adsorbate-slab (adslab) system with an AFM-initialized slab (black), with adsorption energies of −2.28 eV and −1.96 eV, respectively. Distances to the Nitrogen adsorbate are indicated in blue. The adsorbate-induced spin effect primarily arises from nonlocal atoms, leading to lower adsorption energies. A full list of magnetic moments is provided in *SI Appendix*, Table S5.

We proceeded to use our model in order to perform “spin-flipping” inside the slab lattice, as part of Monte Carlo and particle-swarm simulations (*Methods*), with a view to finding a lower-energy spin configuration. For CrN, the spin configuration that is found using our model leads to a lower total slab energy: the AFM spin configuration and FM spin configuration are 2.63 and 3.34 eV higher, respectively, relative to the ML-derived spin configuration [Fig. 6 *A* and *B*). In the example of Fe_2_O_3_, we also observed lower energy in the ML-derived spin configuration (*SI Appendix*, Fig. S13)] than the AFM (0.35 eV) or FM (3.08 eV) spin configurations, albeit less substantially (*SI Appendix*, Fig. S10): It appears that one cannot do much better than AFM ordering on the Fe ions but the particle swarm optimization (PSO) manages to squeeze an additional ∼0.35 eV from optimizing the ordering on the O ions.

In order to demonstrate the impact that different initial magnetic orderings can have on a standard catalysis workflow for computing adsorption energies, we place a nitrogen atom at the same Cr vacancy site across the FM, AFM, and ML-informed (Fig. 6*C*) relaxed clean slab orderings and allow these geometries to optimize in VASP. We compute the adsorption energies to be 1.00 eV, −1.96 eV, and 0.60 eV, respectively. A similar exercise on Fe_2_O_3_, with an oxygen on-top adsorbate on an undercoordinated Fe site, yields similar conclusions: The adsorption energies for the FM, AFM, and ML-informed configurations are 4.43 eV, 5.45 eV, and 4.35 eV, respectively. Not having access to the most stable ML-obtained ordering or initializing surfaces in the FM configuration out of convenience could thus potentially lead to large errors in the adsorption energies, consistent with (23, 41).

Furthermore, we probe adsorbate-induced spin effects by performing spin-flipping for the adsorbate-slab (adslab) configuration, with a view to finding an even lower spin configuration. Intriguingly, for the CrN AFM spin configuration, we are able to find an even lower adslab spin configuration, with an energy reduction of 0.32 eV via the MLIP-powered MC spin flipping simulations (Fig. 6*D*). In this lower-energy adslab spin configuration, we observe substantial changes in spin magnitudes and orientations for nonlocal atoms, such as those located 4.39 Å and 5.33 Å from the nitrogen adsorbate, as well as bottom-layer Cr atoms (see *SI Appendix*, Table S5 for the full list of magnetic moments). This finding highlights the nonlocal spin impact of the adsorbate on electron density, which cannot be readily revealed through DFT local geometry optimization without a good initial guess.

## Discussion

In this work, we demonstrate that incorporating explicit spin degrees of freedom into uMLIPs is of great importance, extending the scope of traditional uMLIPs to study magnetic materials. The proposed method is analogous to nonconstrained spin-polarized DFT calculations, starting from an initial guess to predict (local) equilibrium magnetic moments and associated magnetic ordering.

We found a data quality issue in the MP dataset caused by suboptimal DFT computational settings, which led to nonconverged magnetic moments and subsequently inconsistent labels. This depicts a classic “chicken-and-egg” problem, for which we provide an effective solution by combining our uMLIPs with an anomaly detection approach to refine the dataset. In this regard, data quality should receive more attention in the community when generating datasets in a high-throughput manner. In the same vein, we currently face a data quantity issue, with sparse magmom labels across the PES compared to traditional labels like the energy and forces (42, 43).

Although our model can be naturally applied to study finite-temperature magnetic properties, in connection with MD-MC (11, 14) or disordered local moment molecular dynamics (12, 44), it is limited in its treatment of spin–lattice dynamics (45, 46), where noncollinear-based models and datasets would be more appropriate (11, 16, 43, 47).

## Methods

### DFT Computational Details.

As we discuss in *Unveiling Data Quality Issues*, we had to validate a subset of the dataset for which our trained ML model’s embeddings hinted at anomalous associated magnetic moment labels. For those calculations, we wrote a script to query and write the input sets as they were defined from the Materials Project (48). The only parameter that was subsequently mutated was the NELM parameter, which was set to 1,000 in order to increase the probability of electronic convergence. Additionally, we also used newer pseudopotentials available at NERSC in conjunction with the 6.3.2 VASP binary.

In order to sample the PES at various magnitudes of the magnetic moment on the Gd atom (refer to *Applications*), with a view to understanding the significance of the magnitude of the initial guess, we forced specific spin configurations using the NUPDOWN parameter, ranging from 0 to 8. In addition to that constraint, electronic convergence was still enforced.

In instances where we were not trying to reproduce and validate some of the outlier samples we previously identified, all DFT periodic boundary calculations were performed within the spin-polarized formalism as implemented in the Vienna Ab-initio Simulation Package (VASP-6.3.2), and using the GGA PBE functional (49, 50). Ionic cores were described with the projector augmented wave (PAW) pseudopotentials (51, 52) and the valence electrons were represented through a plane-wave basis set with a kinetic energy cut-off of 500 eV. A (50/a, 50/b, 50/c) and (30/a, 30/b, 1) Γ-centered k-point mesh (53) was employed to describe the first Brillouin zone for bulks and surfaces, respectively. These settings were found to provide a good compromise between accuracy and computational time. The energy convergence criterion was fixed to 10^−4^ eV for the electronic structure, while the Hellman-Feynman forces criterion for geometry relaxation was set to 0.05 eV ${Å}^{-1}$. These specifications were implemented as part of a custom class inheriting from the *MVLSlabSet* input set class of *Pymatgen* (6). As a result, we are able to standardize, version control, and memorialize the VASP parameters across our various clusters and jobs. The rotationally invariant implementation of the Hubbard-U model by Dudarev (54) was employed to handle strongly correlated electrons and the magnitude of the U value applied was in line with the suggestions made by the Materials Project (48). *WhereWulff* (55) was used to launch and manage the calculations.

### Machine-Learning Architecture.

Traditional Message Passing Neural Networks (MPNNs) for molecules operate on undirected graphs G with nodes V and edges E representing atoms and chemical bonds. The atom embedding for each node $h_{i}$ is updated based on a message passing phase that considers its neighbors *j*. This process can be formulated as

[1] $$\begin{array}{c} h_{i}^{t+1}=f_{\text{update}}\left(h_{i}^{t},\sum_{j\in N_{i}}f_{\text{int}}\left(h_{j}^{t},e_{\mathrm{ij}})\right)\right), \end{array}$$

where $e_{\mathrm{ij}}$ represents the edge features between nodes *i* and *j*. The functions *f*_update_ and *f*_int_ are the update function and interaction function (also known as the message function), respectively. SchNet closely follows this formulation with a continuous filter operating on the edge feature, i.e., pairwise distance expanded through radial basis functions $e_{\text{RBF}}\left(r_{\mathrm{ij}}\right)$.

As an extension to the undirected graph, Gemnet-OC introduces directional embeddings, known as edge embeddings, which are updated based on a geometric message passing scheme that is based on quadruplets of atoms—two atoms are interacting (*j* and *k*) and two atoms define the directions (*i* and *l*). The update of $m_{\mathrm{ij}}$ based on $m_{\mathrm{lk}}$ is illustrated as follows:

[2] $$\begin{array}{c} \begin{array}{cc} m_{\mathrm{ij}}^{t+1} & =f_{\text{update}}(m_{\mathrm{ij}}^{t},\sum_{\begin{array}{c} k\in N_{j}\setminus \left\{i\right\}\\ l\in N_{k}\setminus \left\{j,i\right\} \end{array}}f_{\text{int}}(m_{\mathrm{lk}}^{t},e_{\text{RBF}}\left(r_{\mathrm{lk}}\right),\\ & e_{\text{CBF}}\left(r_{\mathrm{lk}},\phi_{\mathrm{jkl}}\right),e_{\text{SBF}}\left(r_{\mathrm{lk}},\phi_{\mathrm{jkl}},\theta_{\mathrm{ijkl}}\right))), \end{array} \end{array}$$

Here, $e_{\text{RBF}}$, $e_{\text{CBF}}$, and $e_{\text{SBF}}$ refer to radial basis function, circular basis function, and spherical basis function, respectively. $r_{\mathrm{lk}}$, $\phi_{\mathrm{jkl}}$ and $\theta_{\mathrm{ijkl}}$ are geometric information representing distances, angles between neighboring edges, and dihedral angles formed by triplets of edges.

The directional message embedding $m_{\mathrm{ij}}^{1}$ were initially generated from the atom embedding $h_{i}^{0}$, $h_{j}^{0}$ and distance via:

[3] $$\begin{array}{c} m_{\mathrm{ij}}^{1}=\mathrm{mlp}\left(h_{i}^{0}\|h_{j}^{0}\|e_{\text{RBF}}\left(r_{\mathrm{ij}}\right)\right) \end{array}$$

Here, mlp denotes multilayer perceptron and ‖ is concatenation.

In the spin-informed GNN framework, atom embedding $h_{i}^{0}$ generated from both element identity embedding $e_{z}$ and atom spin embedding $e_{s}$ through:

[4] $$\begin{array}{c} h_{i}^{0}=\mathrm{mlp}\left(e_{z}\|e_{s}\right) \end{array}$$

To preserve spin-inversion symmetry in energy prediction, $e_{s}$ is generated through feature augmentation that processes both the atomic spin coordinate and its inversion using shared *mlp* parameters, followed by averaging as follows:

[5] $$\begin{array}{c} e_{s}=\left(mlp\left(\hat{s},s_{init.}\right)+mlp\left(-\hat{s},s_{init.}\right)\right)/2 \end{array}$$

In Eq. **5**, $s_{init.}$ is the initial guess (scalar) and $\hat{s}$ is the atomic spin coordinate (unit vector). We use a threshold of 0.1 *μ*_B_ to determine the sign and to filter out noise in the magnetic moment label caused by the ambiguity in assigning spins during electronic structure calculations. This threshold can also be learned from training data using a classifier (Fig. 1*A*), which eliminates the need to predefine it and allows for an end-to-end model.

After certain interaction blocks i.e., message passing layers, the final atomic embedding and edge embedding were obtained from all prior interaction blocks via:

[6] $$h_{i}=\;\text{mlp}\left(h_{i}^{1}\|h_{i}^{2}\|\ldots \|h_{i}^{t}\right),$$

[7] $$m_{ij}=\;\text{mlp}\left(m_{ij}^{1}\|m_{ij}^{2}\|\ldots \|m_{ij}^{t}\right)$$

and based on these embeddings, to preserve physical symmetry, the energy, magmom, direct force predictions were made via:

[8] $$E_{\text{tot}}=\frac{1}{N}\sum_{i}^{N}\text{mlp}(h_{i})$$

[9] $$M_{i}=\;\text{mlp}(h_{i})\hat{s}$$

[10] $$f_{i}=\underset{\begin{array}{c} j\in N_{i} \end{array}}{\sum }\;\text{mlp}(m_{ij})\;{\hat{r}}_{ij}$$

Alternatively, the energy-conserving force can also be derived from

[11] $$\begin{array}{c} f_{i}=-\frac{\partial E_{\text{tot}}}{\partial x_{i}}, \end{array}$$

where ${\hat{r}}_{\mathrm{ij}}$ and $x_{i}$ are edge vector and atomic Cartesian coordinates. Training details and hyperparameters are provided in *SI Appendix*, section S2. For additional details on GemNet-OC and spin-distance edge GNN, readers are directed to the original publications (16, 27).

Our primary design focus is the incorporation of the spin degrees of freedom and the preservation of physical symmetry within the architecture. Specifically, the initial guess and atomic spin coordinates are included in the atom embeddings, which are then used to predict energy and atomic magnetic moments. The atomic spin coordinates guide the prediction of atomic magnetic moments, helping the model focus on learning their magnitude. Force predictions can be made through either direct force prediction (56) or energy-conserving forces. The former relies on edge embeddings, while the latter is derived from autodifferentiation.

Physical symmetries are maintained at the prediction level. Rotationally equivariant predictions, such as direct force predictions, are managed by projecting the embedding scalar onto the edge distance vector (Eq. **10**). Invariant predictions, such as energy and magnetic moments relative to atomic positions, are inherently preserved by the design choice (i.e., relying on distances and angles). Spin-inversion symmetry, which ensures energy consistency with respect to spin configuration, is achieved through feature augmentation (Eq. **5**). This involves processing the atomic spin coordinate and its inversion separately, followed by averaging. Meanwhile, projecting the embedding scalar onto the spin coordinate is used to break spin-inversion symmetry for magnetic moment predictions (Eq. **9**).

The approach of preserving physical symmetry introduced in this work is more suitable for invariant GNNs. In contrast, equivariant GNNs maintain physical symmetry at the representation level. However, it remains questionable whether it is advantageous to preserve symmetry at the representation level, given that equivariant GNNs are generally challenging to train because of expensive tensor product operations and poor optimizer dynamics (57, 58).

### Anomaly Detection.

The anomaly detection method in this work aims to identify samples that deviate from the regular rule of the dataset, due to technical issues such as nonconverged SCF cycles, convergence to metastable spin states caused by poor spin initialization (see examples in Fig. 4*B*), or the use of outdated PSPs and VASP versions (see examples in *SI Appendix*, Table S6). This approach utilizes a GMM and OOD detection method. The GMM groups similar atomic embeddings, while the OOD detection identifies anomalous labels that deviate from the domain within the same group.

A GMM is a probabilistic model that assumes data points are generated from a mixture of several Gaussian distributions with unknown parameters (59). To construct the GMM, we first extract learned atom embeddings from a trained model before the linear projection to the per-atom magmom prediction. The GMM is then fitted using the Expectation–Maximization (EM) algorithm, with weights, means, and precisions initialized by k-means clustering (59, 60). We use a full covariance matrix for each Gaussian, meaning each covariance matrix is full rank and not shared between Gaussians. The number of Gaussians, i.e. groups, is selected using the Bayesian Information Criterion (BIC) (61).

Based on the groups identified by the GMM, we employ the z-score method to detect OOD samples resulting from anomalous labels within each group. The z-score is a statistical measure that quantifies the distance between a data point and the mean of a dataset, expressed in terms of SDs (62). This indicates how many SDs a data point is from the mean of the distribution. The closer a z-score is to 0, the nearer the corresponding data point is to the dataset’s mean. We set thresholds at −5 and 5, identifying data points with z-scores beyond these values as anomalies. This inductive bias, whereby embedding representations that are similar showcase similar labels (10) is at the heart of many bias correction algorithms (36).

These identified anomalies should then be carefully recomputed using DFT settings tailored to address the specific technical issues. As such, developing an automated workflow capable of categorizing the origin of anomalies would be highly beneficial.

### Magnetic Configuration Optimizations.

To identify stable magnetic configurations, which are essential for both determining ground state magnetic ordering and molecular dynamics studies, we have integrated our spin-informed GNN model with various optimization algorithms. The choice of optimization algorithm depends on the specific objectives of the optimization. For optimizing spin vectors alone, we have employed a Monte Carlo spin-flip method. Additionally, to optimize both spin vectors and magnitudes, we have implemented PSO.

The Monte Carlo spin-flip method utilized in this study is based on the Metropolis–Hastings algorithm, a Markov Chain Monte Carlo (MCMC) technique employed for sampling from probability distributions. In each iteration of the Monte Carlo simulation, the spin magnitudes are held constant while a randomly selected spin vector is flipped. The acceptance of the new spin configuration is determined by its predicted energy, evaluated with a probability given by the Boltzmann factor, $e^{-\frac{\Delta E}{k_{B}T}}$, where −ΔE represents the change in energy, *k*_*B*_ is the Boltzmann constant, and *T* is the temperature in Kelvins. This probability is compared to a uniformly distributed random number between 0 and 1.

PSO is a population-based optimization algorithm inspired by the social behavior of birds flocking or fish schooling, and it is particularly effective in high-dimensional optimization problems. The key components consist of the positions (*M*), velocities (*V*), and fitness (*E*) of the particles. In our problem, the positions and fitness correspond to the spin vectors and magnitudes for given structure, and the ML predicted energy, with the goal of minimizing this energy. The optimization process begins with the initialization of spin vectors and magnitudes for a swarm of particles, with each particle having 2N parameters, where *N* is the number of atoms in the structure. For each iteration *t*, each particle *i* updates its spin vectors and magnitudes based on its own experience and the experience of neighboring particles. The velocity of each particle is adjusted according to the following equation:

$$\begin{array}{c} V_{i}^{\left(t+1\right)}=\omega V_{i}^{\left(t\right)}+\phi_{p}r_{p}\left(p_{i}^{\left(t\right)}-M_{i}^{\left(t\right)}\right)+\phi_{g}r_{g}\left(g^{\left(t\right)}-M_{i}^{\left(t\right)}\right) \end{array}$$

Here, $p_{i}^{\left(t\right)}$ and $g^{\left(t\right)}$ are the individual best position of *i* and the global best position. *ω*, *ϕ*_*p*_, and *ϕ*_*g*_ denote three hyperparameters known as the inertia weight and cognitive and social coefficients, respectively. *r*_*p*_ and *r*_*g*_ are random numbers uniformly distributed between 0 and 1.

The spin vector and magnitude are then updated based on velocity using

$$\begin{array}{c} M_{i}^{\left(t+1\right)}=M_{i}^{\left(t\right)}+V_{i}^{\left(t+1\right)} \end{array}$$

This iterative process continues until a specified number of iterations is reached or the swarm converges to an optimal solution.

### Dataset Preparation.

The MP-MM dataset was prepared from the MPtrj dataset that was parsed from the September 2022 version of the Materials Project Database. The MPtrj dataset contains some highly unphysical magnetic moments; for example, Cr has a single-point calculation with approximately 17 *μ*_B_. We employ a filter to exclude any magnetic moment labels outside the range of −8 to 8 *μ*_B_. Additionally, the initial magnetic moment labels are extracted from the Materials Project Database using the MP API, and samples without initial magnetic moment labels are also filtered out. The initial magnetic moments include both MP and non-MP initializations, comprising 93.2% and 6.8% of the MP-MM dataset, respectively. The non-MP initializations are taken from the last frame of the first relaxation trajectory in a double-relaxation scheme. The distribution of initial magnetic moments and their effect on model predictions are shown in *SI Appendix*, Figs. S1*B* and S7.

The Eu/Gd subset was extracted from the MP-MM dataset. Samples with atomic magnetic moments in the range of −2 to 2 *μ*_B_ were refined with DFT calculations, for which a 7 *μ*_B_ was used to initialize the magnetic moments of Eu/Gd (refer to *DFT Computational Details*). Out of 234 DFT calculations, 26 calculations failed to converge and were therefore removed from this subset (the distribution shown in *SI Appendix*, Figs. S2 and S3).

## Supplementary Material

Appendix 01 (PDF)

## Data Availability

The DFT calculations that support the findings of this study are available in the GitHub repository at https://github.com/yurisanspeur/DFT_calculations_spin_informed_model (63). The source code necessary to reproduce all experiments is available on GitHub at: https://github.com/Wenbintum/ocp_mag (64). The CHGNet code is available at https://github.com/CederGroupHub/chgnet (65). Additional details on the database, the Spin-informed GNN framework, anomaly detection, and ML applications are provided in *SI Appendix*.
