# Supplementary material for: Spin-informed universal graph neural networks for simulating magnetic ordering
Source: Proc Natl Acad Sci U S A. 2025 Jul 1;122(27):e2422973122. doi: 10.1073/pnas.2422973122 (PMC12260432; doi:10.1073/pnas.2422973122)
Supplement: Supplementary file 1 — Appendix 01 (PDF) [file pnas.2422973122.sapp.pdf]

Supporting Information: Spin-informed universal graph neural networks for simulating magnetic ordering

Wenbin Xu[a,b], Rohan Yuri Sanspeur[*c], Adeesh Kolluru[c], Bowen Deng[b], Peter Harrington[a,b], Steven Farrell[a,b], Karsten Reuter[d], and John R. Kitchin[†c]

[a]National Energy Research Scientific Computing Center, Berkeley, CA 94720, USA
[b]Lawrence Berkeley National Laboratory, Berkeley, CA 94720, USA
[c]Department of Chemical Engineering, Carnegie Mellon University, Pittsburgh, PA 15213, USA
[d]Fritz-Haber-Institut der Max-Planck-Gesellschaft, Berlin, D-14195, Germany

[*]rsanspeu@andrew.cmu.edu
[†]jkitchin@andrew.cmu.edu

Contents

S1 Additional details on datasets . . . **S3**

S2 Additional details on the spin-informed GNN framework . . . **S5**
S2.1 Model training . . . S8
S2.2 Model hyperparameters . . . S8
S2.3 Effect of initial guess . . . S10

S3 Additional details on anomaly detection . . . **S11**

S4 Additional details on ML applications . . . **S13**

S1 Additional details on datasets

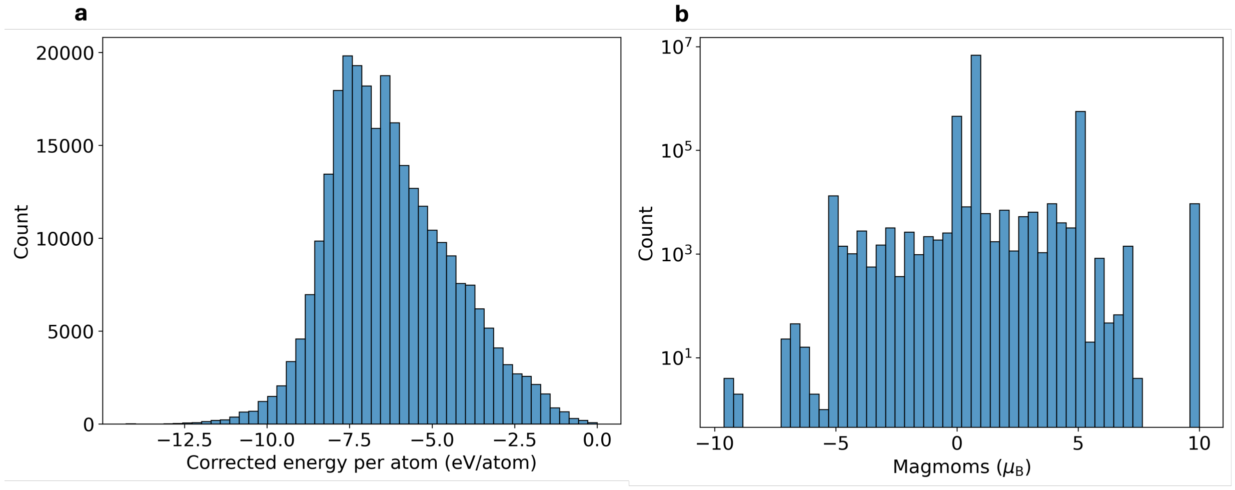

Fig. S1: Distribution of (a) corrected energy per atom and (b) magmom initial guess of MP-MM dataset.

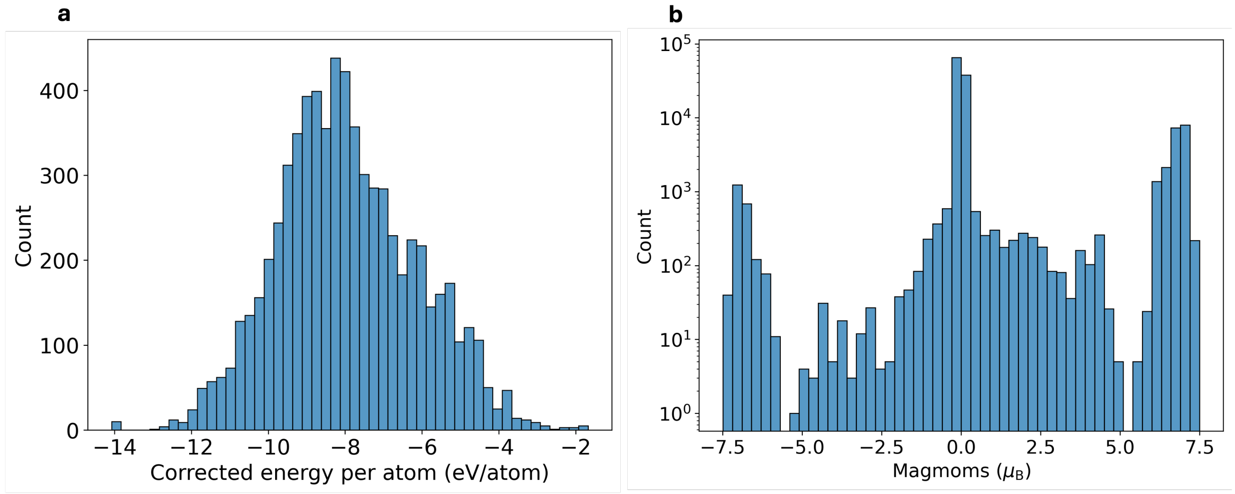

Fig. S2: Distribution of (a) corrected energy per atom and (b) magmom labels of Gd/Eu subset.

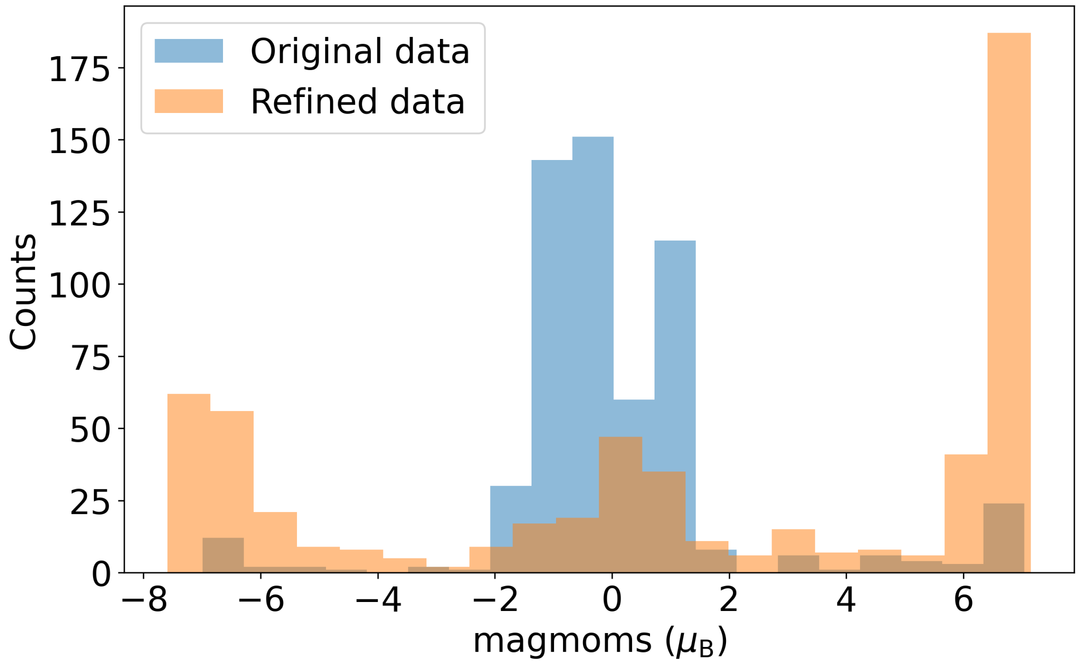

Fig. S3: Distribution of magmom labels for the original (in blue) and refined (in orange) 234 data points, including magmom labels of Gd/Eu in the range of -2 to 2 μ_B. For the refined DFT calculations, the NELM parameter was set to 1000 to increase the probability of electronic convergence, along with the use of the newest pseudopotentials.

S2 Additional details on the spin-informed GNN framework

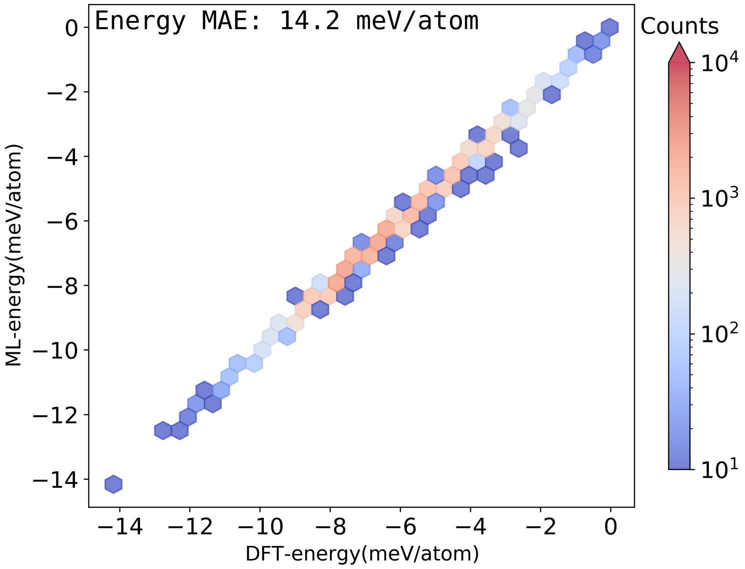

Fig. S4: Parity hexbin plot of DFT-calculated vs. ML-predicted corrected energy per atom for test set of the MP-MM dataset. The color bar denotes the number of points in each hexbin, and only hexbins with more than 3 points are displayed.

	Random init.	Element-based init.	MP init.
Magmoms MAE	0.017	0.010	0.009
On atoms $> 0.1\ \mu_B$	0.082	0.057	0.053

Table S1: The effect of different types of initial guesses on the MP-MM test split, where datapoints with MP initialization are selected, comprising 93.5% of the test split. For random initialization, magnetic moments are sampled randomly from -10 to 10 μ_B. The element-based initialization is computed using the spin-only formula (see Eqs. S1–S2), based on the number of unpaired electrons (n in Eq. S2) in their ground-state electron configuration. The MAE is in μ_B.

$$\mu_s = \sqrt{4S(S+1)}\,\mu_B \tag{S1}$$

$$= \sqrt{n(n+2)}\,\mu_B \tag{S2}$$

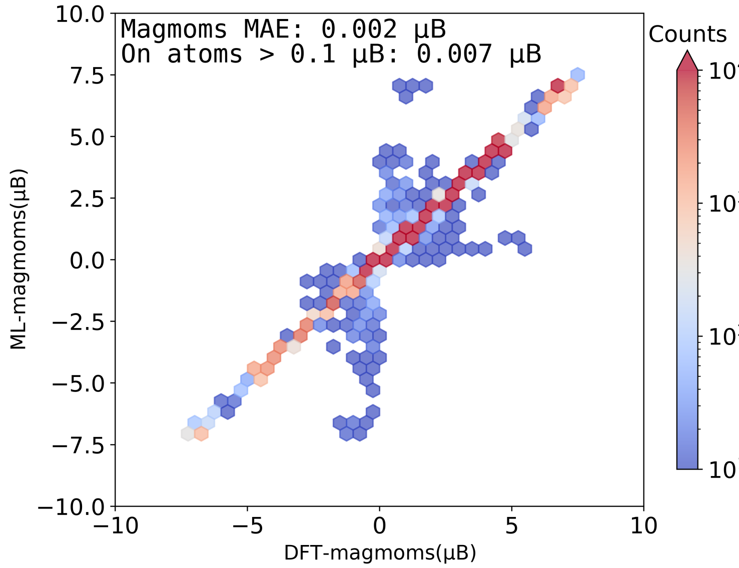

Fig. S5: Parity hexbin plots of DFT-calculated vs. ML-predicted magmoms for training set of the MP-MM dataset. The color bar denotes the number of points in each hexbin, and only hexbins with more than 3 points are displayed.

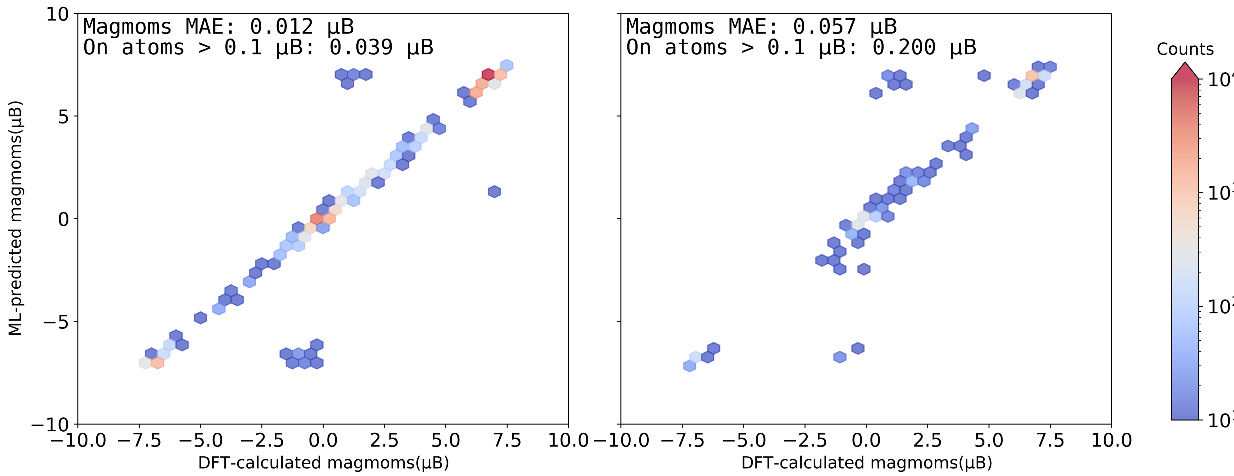

Fig. S6: Parity hexbin plots of DFT-calculated vs. ML-predicted magmoms for (a) training set of the Gd/Eu subset, (b) validation set of the Gd/Eu subset. The color bar denotes the number of points in each hexbin, and only hexbins with more than 3 points are displayed.

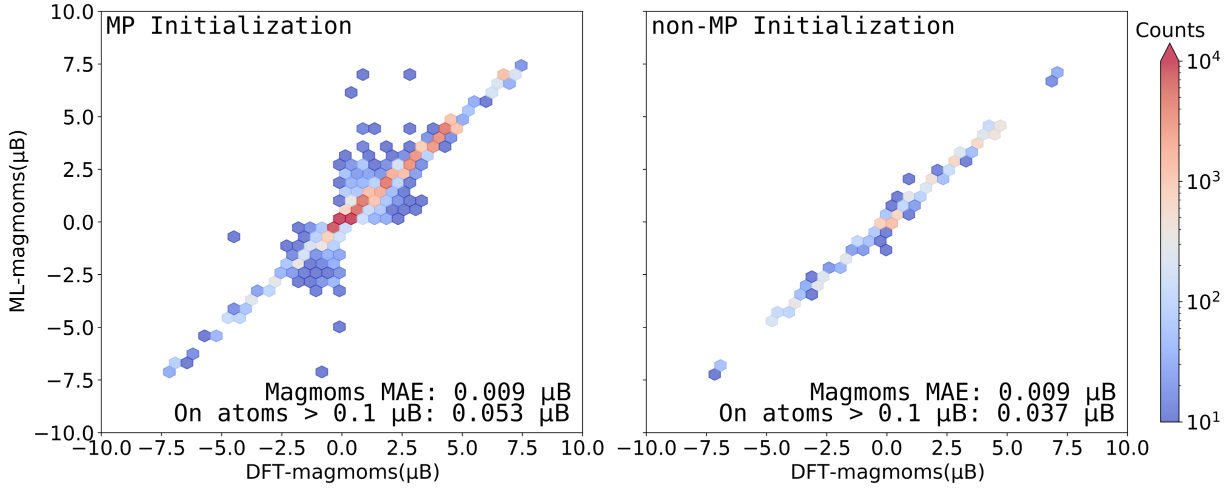

Fig. S7: Parity hexbin plots of DFT-calculated vs. ML-predicted magnetic moments for the MP-MM test split: (a) using MP initialization, comprising 93.5% of the data, and (b) using non-MP initialization, comprising the remaining 6.5%. The color bar denotes the number of points in each hexbin, and only hexbins with more than 5 points are displayed. While the overall MAE remains unchanged at 0.009 μ_{B}, the MAE for atoms with magnetic moments greater than 0.1 μ_{B} improves from 0.053 μ_{B} with MP initialization to 0.037 μ_{B} with non-MP initialization. Additionally, the number of outliers is significantly reduced in the non-MP case. the number of outliers is significantly reduced in the non-MP case. This suggests that a better initial guess can help improve model predictions.

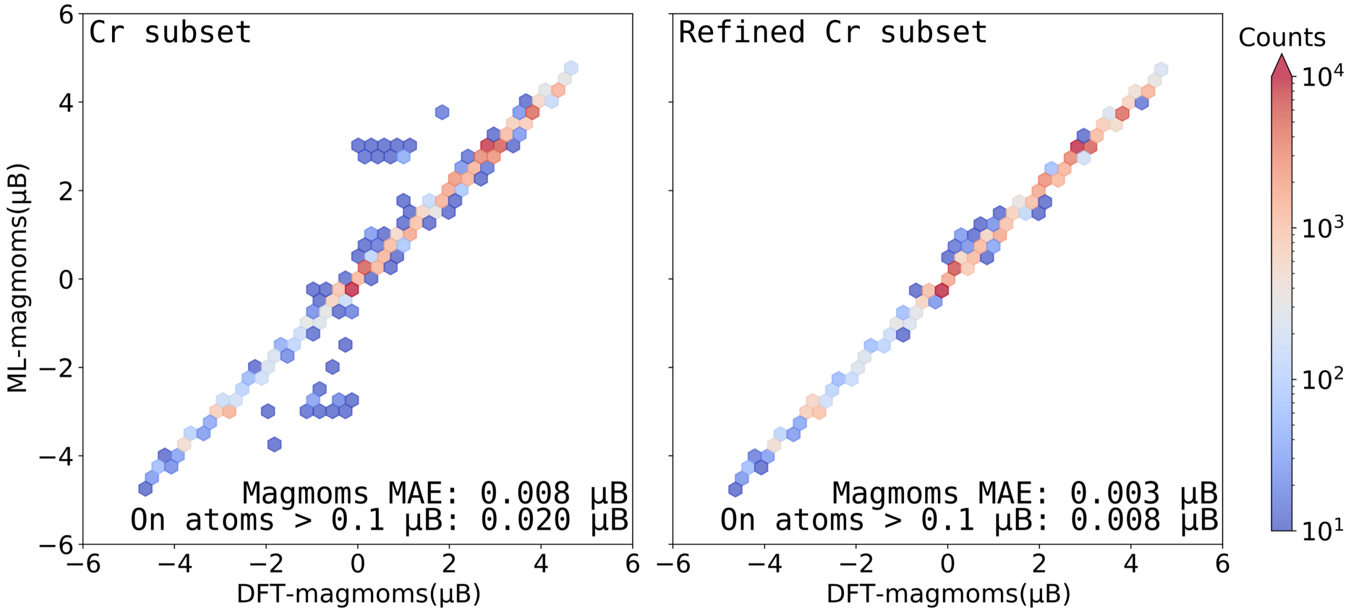

Fig. S8: Parity hexbin plots of DFT-calculated vs. ML-predicted magnetic moments for the training set of the Cr subset, shown both without and with refinement via anomaly detection. The color bar indicates the number of data points per hexbin, and only hexbins containing more than 5 points are displayed.

S2.1 Model training

All ML models were trained to minimize the weighted MAE, with equal weights assigned to tasks predicting energy and magnetic moments. The DFT energies were normalized with elemental reference energies. The CHGNet training protocol followed the approach outlined in [1], where the magnitude of DFT magnetic moments was used for training, while other models considered both magnitude and sign. For the subset, models were trained using 32-bit precision and the Adam optimizer with an initial learning rate of 10^{-3} as well as ReduceLROnPlateau scheduler. To speed up training for the full dataset, 16-bit mixed precision was used along with the FusedLAMB optimizer, implemented with the NVIDIA APEX library. The FusedLAMB optimizer is an advanced algorithm designed to enhance the efficiency and stability of training large-scale machine learning models, which performs gradient clipping as part of its optimization process. All ML models were trained using 1 to 32 NVIDIA A100 GPUs on the Perlmutter supercomputer, which is maintained by the National Energy Research Scientific Computing Center (NERSC) at Lawrence Berkeley National Laboratory (LBNL).

S2.2 Model hyperparameters

Hyperparameters	Values
Hidden channels	128
Filters	128
Interaction blocks	6
Gaussians	50
Cutoff	10
Max neighbors	50

Table S2: SchNet Hyperparameters

Hyperparameters	Values
No. spherical basis	7
No. radial basis	128
No. blocks	4
Atom embedding size	256
Edge embedding size	512
Triplet edge embedding input size	64
Triplet edge embedding output size	64
Quadruplet edge embedding input size	32
Quadruplet edge embedding output size	32
Atom interaction embedding input size	64
Atom interaction embedding output size	64
Radial basis embedding size	16
Circular basis embedding size	16
Spherical basis embedding size	32
No. residual blocks before skip connection	2
No. residual blocks after skip connection	2
No. residual blocks after concatenation	1
No. residual blocks in atom embedding blocks	3
No. atom embedding output layers	3
Cutoff	12
Quadruplet cutoff	12
Atom edge interaction cutoff	12
Atom interaction cutoff	12
Max interaction neighbors	30
Max quadruplet interaction neighbors	8
Max atom edge interaction neighbors	20
Max atom interaction neighbors	1000

Table S3: GemNet-OC Hyperparameters

Hyperparameters	Values
Atom feature size	64
Bond feature size	64
Angle feature size	64
No. radial basis	31
No. angular basis	31
No. blocks	4
Atom conv. size	64
Bond conv. size	64
Angle layer hidden size	0
Conv. dropout	0
MLP hidden size	64,64,64
Atom graph_cutoff	6
Bond graph cutoff	3
Cutoff coeff.	8

Table S4: CHGNet Hyperparameters

S2.3 Effect of initial guess

In the MP-MM dataset, 6.8% of the data originate from non-MP initializations, while 93.2% stem from MP initializations. The non-MP initializations are taken from the last frame of the first relaxation trajectory in a double relaxation scheme, which provides a better starting point that is closer to the ground truth. To explore the effect of non-MP initialization, we present the corresponding parity plot and accuracy metrics in Fig. S7. As seen, while the overall mean absolute error (MAE) remains unchanged at 0.009 μ_{B}, the MAE for atoms with magnetic moments greater than 0.1 μ_{B} improves from 0.053 μ_{B} (MP initialization) to 0.037 μ_{B} (non-MP initialization). Additionally, the number of outliers is significantly reduced in the non-MP case. This suggests that a better initial guess can help improve model predictions. It should also be noted that, in order to train an MLIP that reliably captures the many-to-one mapping in collinear magnetic systems, initial guesses should be drawn from a more diverse set rather than relying solely on MP initialization.

We then systematically tested different types of initial guesses, namely random initialization and element-based initialization, in comparison with MP initialization. In the random initialization, magnetic moments are sampled randomly from -10 to 10 μ_{B}. The element-based initialization is computed using the spin-only formula (see Eqs. S1–S2), based on the number of unpaired electrons (n in Eq. S2) in their ground-state electron configuration. As expected, better initial guesses lead to improved predictive performance, as shown in Tab. S1. Element-based initialization performs slightly worse than MP initialization, as it has only a single element–value pair without accounting for different oxidation /ionic states. Random initialization performs significantly worse than both.

Given the nature of the many-to-one relationship, it is advantageous from the perspective of initial guesses to start with values closer to the ground truth. This can be facilitated through theoretical approximations that are readily computed, such as the spin-only formula for magnetic moment, while also accounting for different oxidation or ionic states. From the model's perspective, a high-quality dataset with diverse initial guesses is essential for effectively learning this many-to-one relationship. Once trained on such a dataset, the model can be used to optimize magnetic moments via strategies such as Monte Carlo spin-flipping or particle swarm optimization.

S3 Additional details on anomaly detection

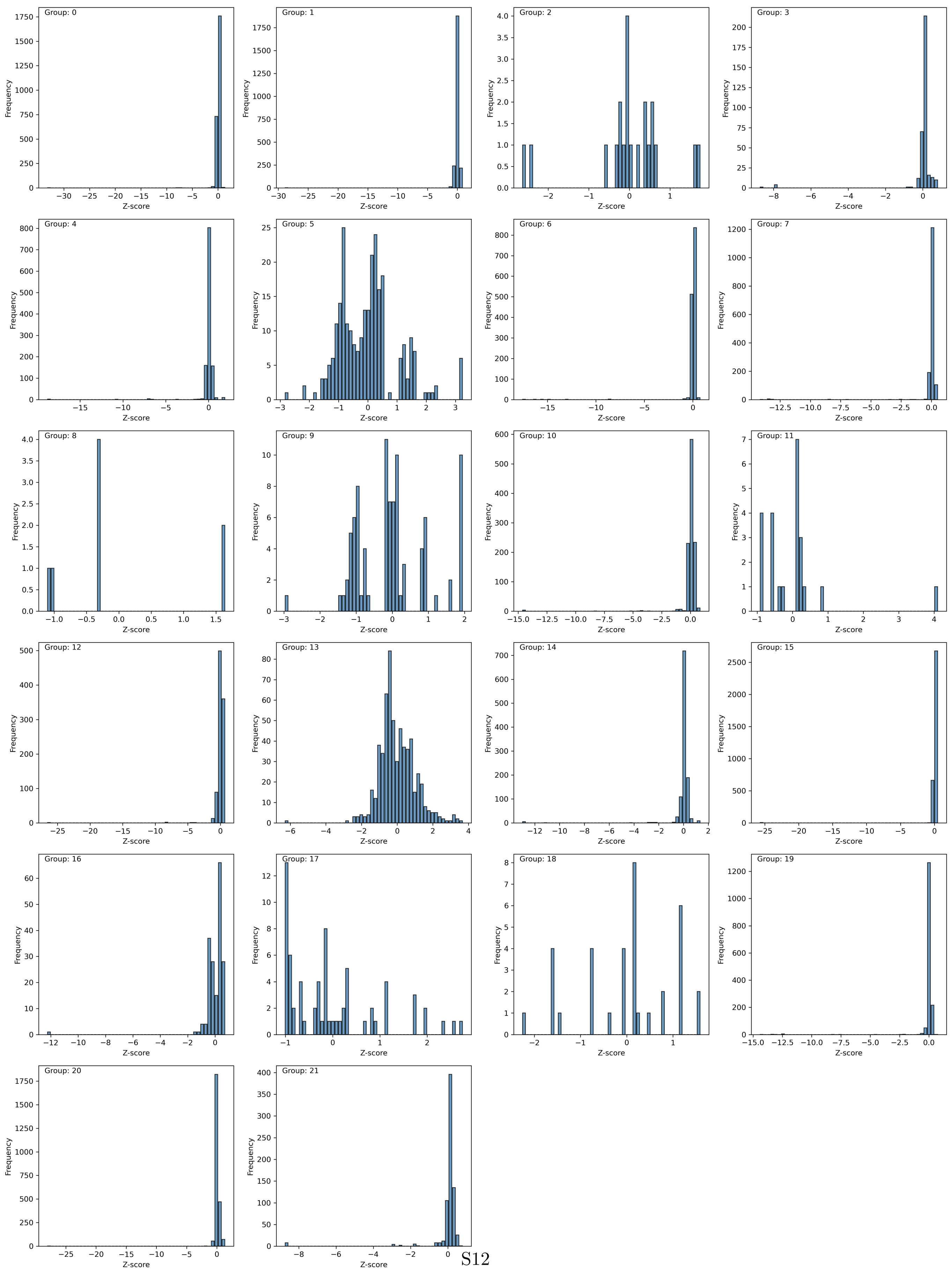

Fig. S9: Distribution of z-scores calculated for each group identified by the GMM model. The closer a z-score is to 0, the nearer the corresponding data point is to the group's mean.

S4 Additional details on ML applications

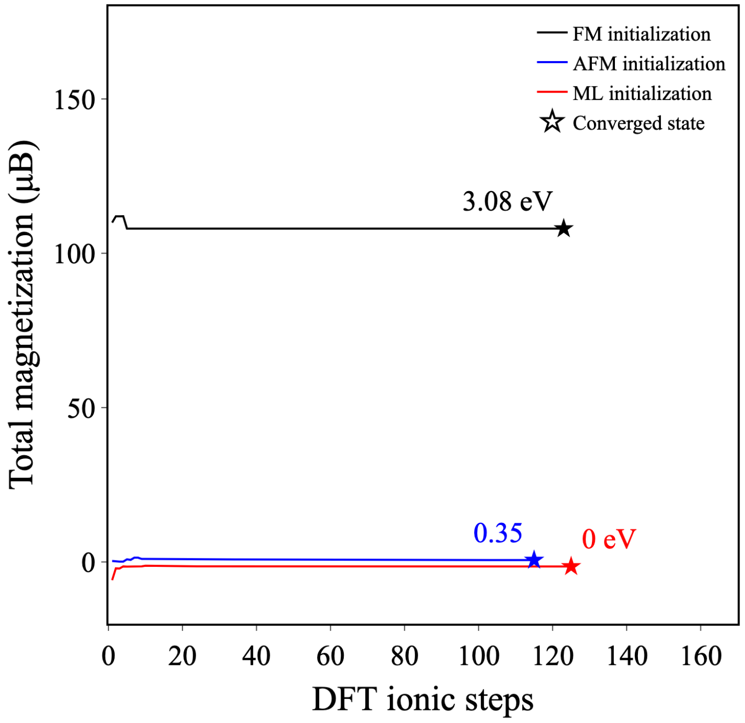

Fig. S10: Plot of total magnetization in μ_B versus ionic steps index for different magnetic ordering initializations of Fe_2O_3.

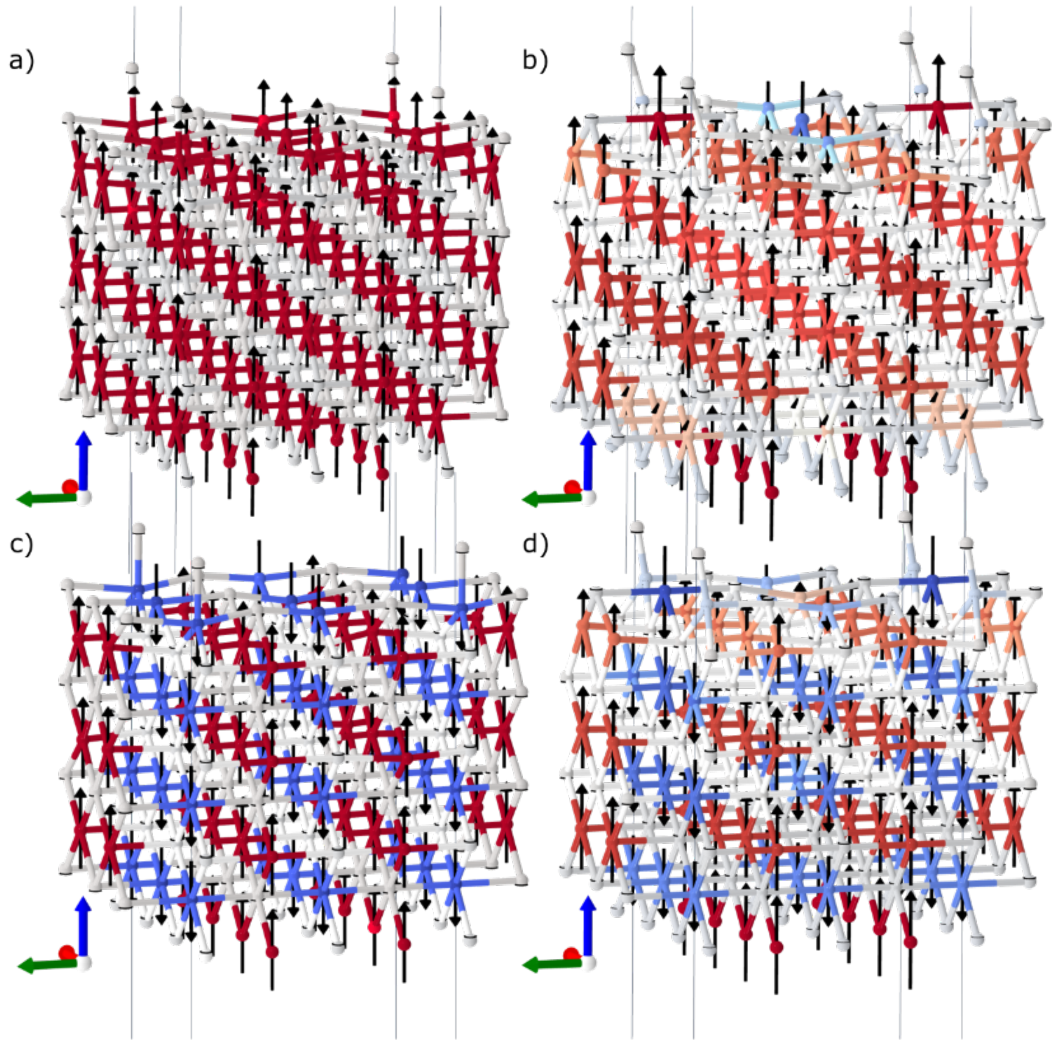

Fig. S11: Shows the structures for the case study that investigates the sensitivity of the adsorption energy to the initial magnetic ordering guess for the clean slab. The adsorption energy is computed by first relaxing the clean slab with the respective ordering. Subsequently, this relaxed geometry is used as the starting geometry with a non-magnetic nitrogen adsorbate placed at a consistent site across the three distinct orderings. The three adslabs are then relaxed with the corresponding initial guess for the magnetic ordering imposed. Panels a) and c) represent the initial configurations for the FM and AFM orderings, respectively, while b) and d) represent the resulting relaxed configurations from VASP. a) $\rightarrow$ b): while most ions are stuck in the FM orientation ($\uparrow$), some of them switch to the spin down ($\downarrow$) state during the course of the relaxation. c) $\rightarrow$ d): the ordering is maintained to a higher extent though the magitude of the magnetic moments attenuate, depicted by the lower shade of colors. The coloring scheme used depicts both the magnetic moment magnitude and direction: red represents spin up ($\uparrow$) and blue represents spin down ($\downarrow$). Magnitude-wise it goes from dark red to dark blue via white for non-magnetic ions. Crystal tookit [2] was used to render the magnetic orderings but with modifications to internal parameters for easier characterization.

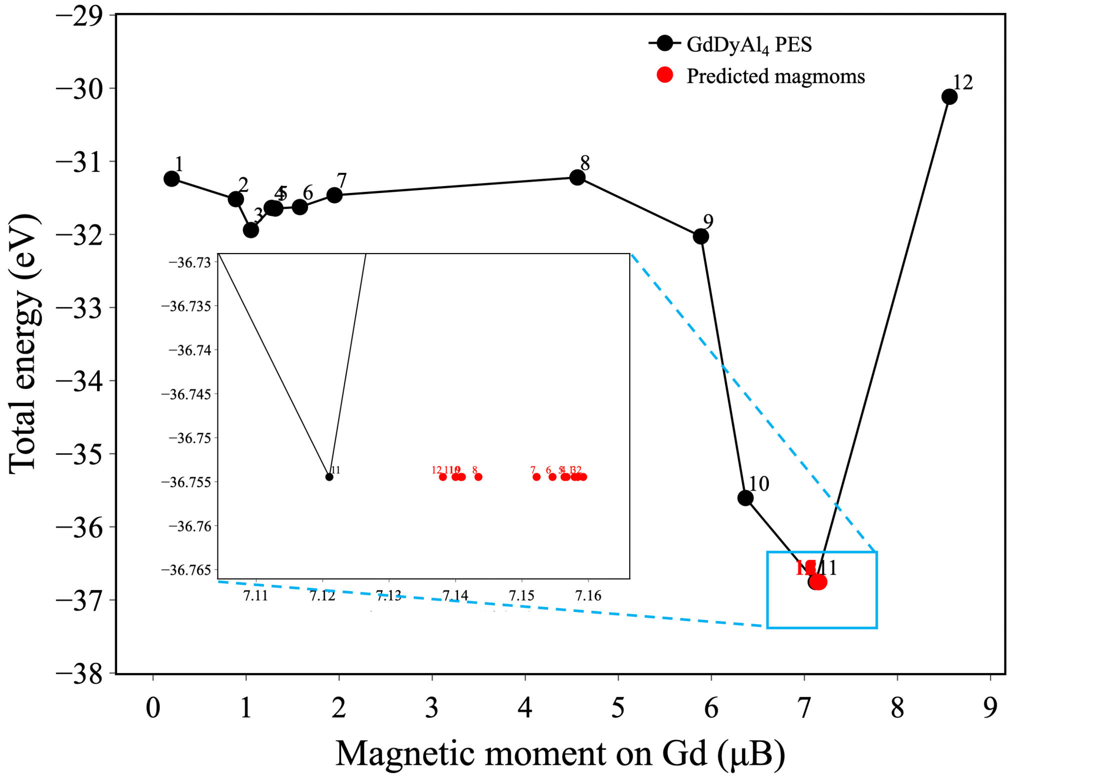

Fig. S12: Schematic of the potential energy surface of $GdDyAl_4$ calculated via VASP (black) and the predicted magnetic moments via SI-GemNet-OC (red). SI-GemNet-OC predicted the converged magnetic moments for given initial spin guesses, with indices illustrating the mapping between the initial guesses and the predicted magnetic moments. All predicted magnetic moments are approximately 7.15 μ_B, demonstrating a clear many-to-one relationship and closely aligning with the DFT-calculated best minimum of 7.12 μ_B. The total energy of the best minimum is used as the y-axis for these predicted points for ease of illustration.

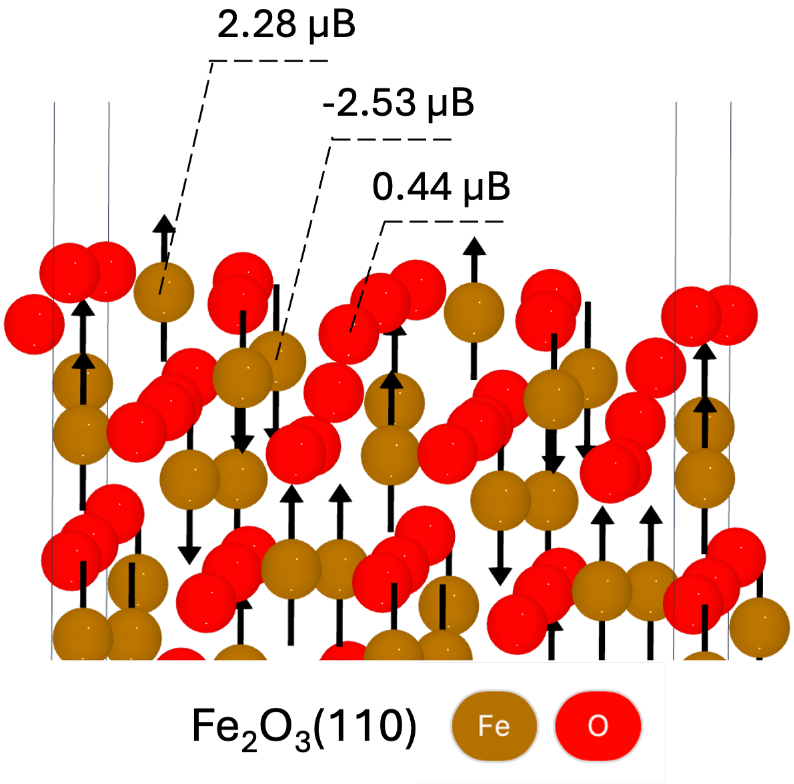

Fig. S13: Spin-informed ML potential is used as a surrogate in particle-swarm spin flipping for surface-related applications. Fe_2O_3 was cut along the (110) plane. Fe is depicted in orange while O is depicted in red. The most stable ordering from the ML-potential aided simulation is FiM, since the total magnetization of the unit cell is not zero. Most of the magnetism is at the level of the Fe ions but we do see some O atoms exhibiting non-negligible magnetic behavior.

Atom index	Adslab	Spin-flipped adslab	Atom index	Adslab	Spin-flipped adslab
0	-1.979	-2.336	33	-0.046	0.066
1	-2.008	-2.353	34	-0.044	0.066
2	-1.984	-2.339	35	-0.045	0.066
3	-2.014	-2.352	36	-0.110	0.207
4	2.842	-2.970	37	-0.100	0.208
5	2.842	-2.970	38	-0.110	0.207
6	2.841	-2.970	39	-0.100	0.208
7	2.842	-2.970	40	0.015	0.036
8	-2.252	-2.322	41	0.014	0.035
9	-2.212	-2.307	42	0.017	0.038
10	-2.278	-2.342	43	0.017	0.039
11	-2.234	-2.305	44	-0.034	0.011
12	2.455	2.240	45	-0.029	0.013
13	2.449	2.236	46	-0.034	0.011
14	2.455	2.240	47	-0.029	0.012
15	2.452	2.236	48	0.009	0.005
16	-2.299	-2.290	49	0.021	0.017
17	-1.921	-1.978	50	0.009	0.004
18	-2.358	-2.346	51	0.011	0.004
19	-2.001	-1.948	52	-0.015	-0.007
20	2.346	2.348	53	-0.023	-0.020
21	2.362	2.369	54	-0.016	-0.008
22	2.349	2.352	55	-0.019	-0.017
23	2.364	2.369	56	-0.132	-0.161
24	-0.376	-0.348	57	-0.012	-0.060
25	-1.081	0.525	58	-0.035	-0.021
26	-2.705	-2.691	59	-0.030	-0.014
27	0.666	-0.032	60	0.029	0.028
28	1.879	1.899	61	-0.013	-0.022
29	1.952	1.944	62	0.023	0.023
30	1.924	1.935	63	-0.013	-0.024
31	1.969	1.961	64	0.025	0.032
32	-0.045	0.066			

Table S5: Magnetic moments of individual atoms in the CrN adslab and the adsorbate-induced spin-flipping adslab. The CrN adslab was relaxed via DFT with an AFM-initialized clean surface. The adsorbate-induced spin-flipping adslab was generated through Monte Carlo spin flipping on the CrN adslab, followed by DFT relaxation, resulting in a spin configuration with lower adsorption energy (-2.28 eV vs. -1.96 eV). The adsorbate-induced spin effect primarily arises from non-local atoms highlighted in red.

mp-1385733			mp-1404606		
**Atom index**	**MP cal.**	**New cal.**	**Atom index**	**MP cal.**	**New cal.**
1	-0.008	-0.002	1	3.299	0.259
2	-0.008	-0.004	2	0.036	-0.013
3	0.006	0	3	0.004	-0.002
4	0.006	-0.001	4	0.036	-0.013
5	0	0.002	5	0.004	-0.002
6	0.009	0.001	6	-0.02	-0.043
7	3.705	0.16	7	-0.064	-0.018
8	-0.076	0.046	8	-0.064	-0.018

Table S6: Comparison of MP and new calculations using latest PSPs potpaw_PBE.64 and VASP 6.4.3 for two exemplified anomalies in Cr subset that are identified by anomaly detection method. The new calculations result in lower energies and converge to different magnetic moments highlighted in red. The magnetic moment is in μ_{B}.

References

1. Deng, B. *et al.* CHGNet as a pretrained universal neural network potential for charge-informed atomistic modelling. *Nat. Mach. Intell.* **5,** 1031–1041 (2023).
2. Horton, M. *et al.* Crystal Toolkit: A Web App Framework to Improve Usability and Accessibility of Materials Science Research Algorithms. *arXiv preprint arXiv:2302.06147* (2023).
